# 4039 The Development of a Mentored Writing Workgroup

**DOI:** 10.1017/cts.2020.224

**Published:** 2020-07-29

**Authors:** Bernadette Capili, Bernadette Capili, Jeanne Walker, Barry Coller, Kate Brown

**Affiliations:** 1 The Rockefeller University

## Abstract

OBJECTIVES/GOALS: The purpose of the mentored writing workgroup developed at The CCTS at the Rockefeller University (CCTS RU) was to promote scholarship among clinical research nurses (CRN). The goal was to publish and/or present their projects at national professional association meetings. METHODS/STUDY POPULATION: A two-part writing workshop was presented at RU in December 2018 and January 2019. Members of the RU nursing staff and CRNs from local institutions were invited to attend. Twenty-four CRNs participated in the workshops. The first workshop focused on the writing process, styles of writing and how to get started. The second concentrated on components of a manuscript, categories of papers, selection of the journal, and communications with the editorial team. After the workshops, the CRNs from RU was offered the opportunity to participate in the mentored writing workgroup. To participate, the CRN had to agree to attend scheduled writing meetings and submit written work for each session. The scheduled submission of written materials ensured the CRN was committed to completing their manuscripts or presentations. RESULTS/ANTICIPATED RESULTS: Three CRNs from RU participated in the writing workgroup. Two papers were accepted for publication, and one manuscript is under-review, two abstracts accepted by an international professional organization, and two presentations conducted at an area nursing school and medical center. DISCUSSION/SIGNIFICANCE OF IMPACT: A mentored writing workgroup wherein participants commit to attend writing meetings and to submit written materials in a scheduled-matter can promote scholarship. CONFLICT OF INTEREST DESCRIPTION: NA.

